# Estimation of the reproduction number of influenza A(H1N1)pdm09 in South Korea using heterogeneous models

**DOI:** 10.1186/s12879-021-06121-8

**Published:** 2021-07-07

**Authors:** Yunjeong Lee, Dong Han Lee, Hee-Dae Kwon, Changsoo Kim, Jeehyun Lee

**Affiliations:** 1 Department of Computational Science and Engineering, Yonsei University, 50, Yonsei-ro, Seoul, 03722 South Korea; 2 Korea Disease Control and Prevention Agency, 187, Osongsaengmyeong 2-ro, Cheongju-si, 28159 South Korea; 3 Department of Mathematics, Inha University, 100, Inha-ro, Incheon, 22212 South Korea; 4 Department of Preventive Medicine and Public Health, Severance Hospital, Yonsei University College of Medicine, 50-1, Yonsei-ro, Seoul, 03722 South Korea; 5 Department of Mathematics, Yonsei University, 50, Yonsei-ro, Seoul, 03722 South Korea

**Keywords:** Influenza, Reproduction number, Antiviral agents

## Abstract

**Background:**

The reproduction number is one of the most crucial parameters in determining disease dynamics, providing a summary measure of the transmission potential. However, estimating this value is particularly challenging owing to the characteristics of epidemic data, including non-reproducibility and incompleteness.

**Methods:**

In this study, we propose mathematical models with different population structures; each of these models can produce data on the number of cases of the influenza A(H1N1)pdm09 epidemic in South Korea. These structured models incorporating the heterogeneity of age and region are used to estimate the reproduction numbers at various terminal times. Subsequently, the age- and region-specific reproduction numbers are also computed to analyze the differences illustrated in the incidence data.

**Results:**

Incorporation of the age-structure or region-structure allows for robust estimation of parameters, while the basic SIR model provides estimated values beyond the reasonable range with severe fluctuation. The estimated duration of infectious period using age-structured model is around 3.8 and the reproduction number was estimated to be 1.6. The estimated duration of infectious period using region-structured model is around 2.1 and the reproduction number was estimated to be 1.4. The estimated age- and region-specific reproduction numbers are consistent with cumulative incidence for corresponding groups.

**Conclusions:**

Numerical results reveal that the introduction of heterogeneity into the population to represent the general characteristics of dynamics is essential for the robust estimation of parameters.

## Background

The reproduction number is defined as the average number of secondary cases generated by a typical primary case. It is a measure of the transmission potential associated with the contact rate, duration of infectivity, and probability of transmission per contact. The maximum reproduction number is attained when an infectious person is introduced into a totally susceptible population and is called the basic reproduction number, *R*_0_. Various approaches such as the exponential growth rate of infections during the early epidemic stage, model-based schemes, and maximum-likelihood estimations have been used to analyze this number [1–4].

When an infection spreads throughout a population, the time-dependent effective reproduction number, *R*_*t*_, is often more useful for assessing the transmission potential throughout a pandemic, especially during the period with the highest level of activity. Real-time estimation continues to track the number of secondary infections caused by a single infective, providing a quantitative measure of the time evolution of the epidemic force. Cruz-Pacheco et al. demonstrated the manner in which sanitary measures reduce the prevalence of an infected population [1]. Estimates of the reproduction number were shown to decrease from 1.4-1.5 initially to 1.1-1.2 later in the summer, which was most likely because of the vacation period and the seasonality of influenza transmission [5]. In addition to capturing temporal dynamics, it is important to consider heterogeneous patterns of the transmission. It is well known that school-age children are disproportionately responsible for influenza transmissions. Estimates of the age-specific reproduction number help with our understanding of the role of each group in the transmission dynamics and with devising effective targeting mitigation strategies.

If all incident cases could be traced back to their index cases, estimating the reproduction number would simply be a matter of counting the number of secondary cases. However, with most epidemics, only the epidemic curve is observed, and there is no available information regarding who infected whom. To appropriately estimate the reproduction number from the influenza outbreak data, it is essential that the selected model capture the underlying dynamics embedded in the data. The objective of this study is to estimate the reproduction numbers based on the incidence data.

When the World Health Organization announced the emergence of influenza A(H1N1)pdm09 (pH1N1) in 2009 [6], the first probable patient in South Korea was identified on April 28. A total of 763,759 confirmed cases, of which 270 were fatal, were reported by the end of August 2010 [7]. During the initial epidemic phase, the main control measure was containment through quarantine and isolation. Surveillance programs in schools and medical facilities were implemented, and all confirmed cases were investigated. However, when community outbreaks were detected in June, the intervention policy switched from containment to mitigation, including vaccination and antiviral prescription. Vaccination was started on October 27, 2009, and 12.7 million people were vaccinated by the end of August 2010. Before August 20, antiviral agents were prescribed to patients with acute febrile respiratory illness (AFRI) and who had a history of travel abroad or contact with a confirmed patient. However, when the number of community-acquired cases increased, antiviral agents were prescribed to patients with AFRI symptoms.

**Fig. 1:**
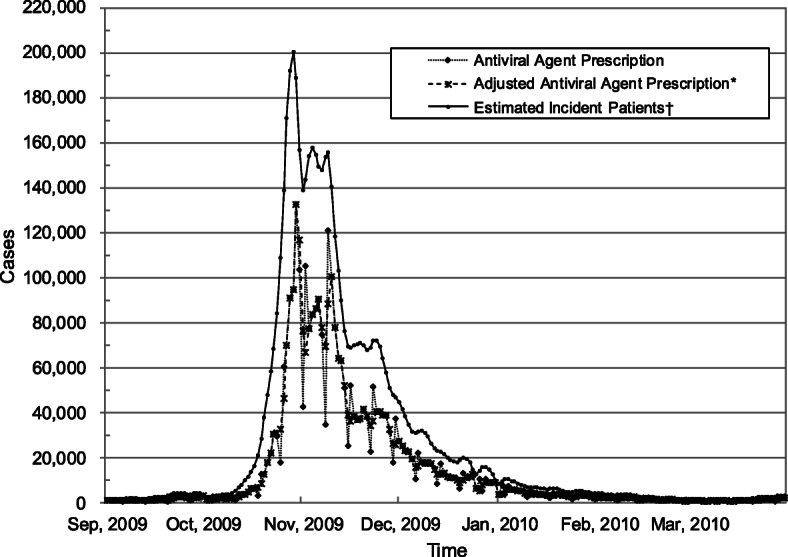
Daily antiviral agent prescription (dotted line) and incident patients (solid line) from September 1, 2009 to March 30, 2010. The incidence data used for model calibration was imported from literature and refer to [8] for details

According to the database, 3,087,788 courses of antiviral agents were prescribed from August 21, 2009 to April 30, 2010. The daily number of incident patients was estimated based on the amount of prescribed antiviral agents (Refer to [8] for details). Figure 1 shows the temporal incidence distribution of pH1N1 in South Korea. The amount of antiviral agents prescribed and the number of incident patients soared from mid-October, reached its peak at the end of October, and started declining in mid-November.

**Fig. 2:**
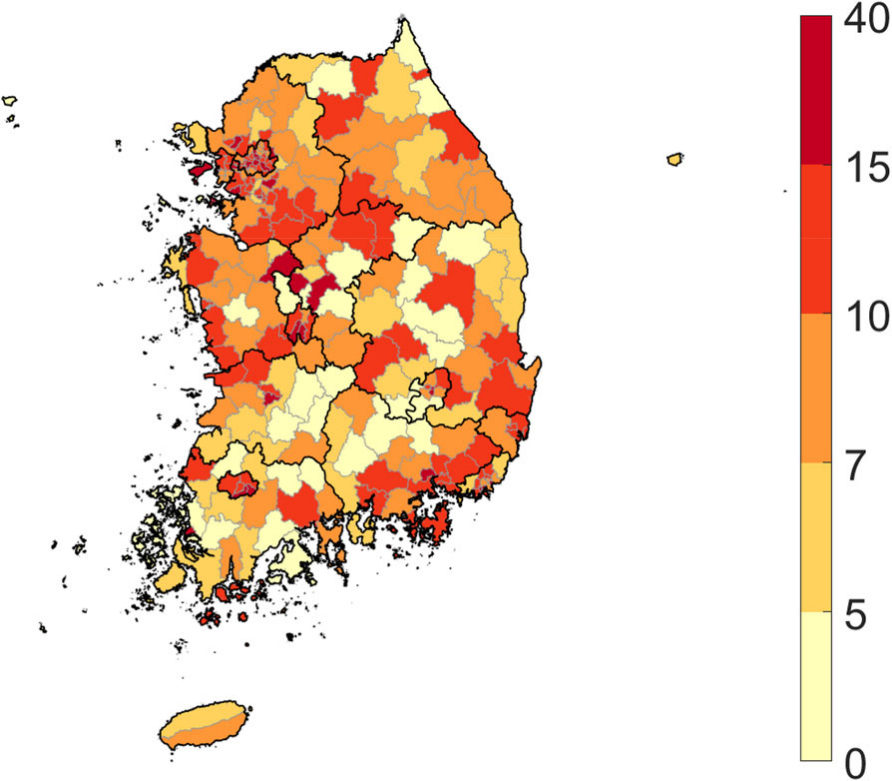
Cumulative incidence by region, which is own figure drawn from the incidence data

Demographic and regional characteristics are illustrated in Table 1 and Fig. 2. The incidence rate is higher in children and students than in other age group individuals (Table 1). The rate is higher in urban areas than in rural areas and is the highest in the national capital and the south-eastern region (Fig. 2).

**Table 1:** Cumulative incidence by age

Age	Population size	Total number of cases	Incidence rate(%)
0-4	2273634	917793	40
5-9	2671496	1084094	41
10-14	3284134	920937	28
15-19	3368818	781238	23
20-24	3153001	305403	10
25-29	4001090	341940	9
30-34	3856438	290354	8
35-39	4466962	303670	7
40-44	4244225	220743	5
45-49	4321262	164735	4
50-54	3761143	149067	4
55-59	2611705	97571	4
60-64	2117041	73540	3
65-69	1870279	66033	4
70+	3306607	100728	3

In this paper, two different structured models are proposed to estimate reproduction numbers on the basis of the epidemic curve. We begin by introducing a basic SIR model to describe a single outbreak and build age- and region-structured models by incorporating population heterogeneity. Numerical simulations are conducted to analyze the impact of terminal time and the effect of heterogeneous structures on the estimation of parameters. Finally, the proposed models are applied to the 2009 incidence data of novel pH1N1 in South Korea to compute the age- and region-specific reproduction numbers.

## Methods

### Basic SIR model

We consider the standard SIR model to represent single-outbreak influenza dynamics. The model classifies individuals into three key compartments: susceptible, infected, and recovered. The nonlinear system of differential equations describing the dynamics is given by the following equation.

$$ \begin{aligned} S^{\prime}(t) &= -\beta S(t)I(t) \\ I^{\prime}(t) &= \beta S(t)I(t) - \gamma I(t) \\ R^{\prime}(t) &= \gamma I(t). \end{aligned} $$

The state variables *S*(*t*),*I*(*t*), and *R*(*t*) denote the number of individuals who are susceptible, infected, and recovered, respectively, at time *t*. The number of contact events sufficient for transmitting an infection during unit time per individual is *βN* based on the mass action incidence. Infective individuals leave the compartment at the recovery rate *γ*, thereby acquiring immunity to the disease. We can drop the equation for *R*(*t*) as it has no effect on the dynamics of others and hence is determined once *S*(*t*) and *I*(*t*) are known. Based on the SIR model, we have the time-dependent net reproduction number *R*_*t*_=*βS*(*t*)/*γ*, which quantifies the level of transmission at time *t*. Note that *R*_*t*_ is the per-infective rate at which new infections occur within the average duration of infection at time *t*.

### Age-structured model

An age-structured model is employed to estimate the reproduction number of pH1N1 because the transmission rate is higher in preschool and schoolchildren than in other age group individuals, in general. We consider a subgroup SIR model where the population is divided into *n*_*a*_ age groups with different transmission dynamics. We denote the number of susceptible and infected individuals within the *i*^*th*^ age group by *S*_*i*_ and *I*_*i*_, respectively. Let *β*_*ij*_ refer to the transmission from the *j*^*th*^ age group to the *i*^*th*^ age group, and *β* =[*β*_*ij*_] denote the transmission matrix, also known as Who-Acquires-Infection-From-Whom matrix. Putting these elements together, we have the following system of differential equations.

1 $$ \begin{aligned} S_{i}^{\prime}(t) &= -\sum_{j=1}^{n_{a}} \beta_{{ij}} S_{i}(t)I_{j}(t) \\ I_{i}^{\prime}(t) &= \sum_{j=1}^{n_{a}} \beta_{{ij}} S_{i}(t)I_{j}(t) - \gamma_{i} I_{i}(t). \end{aligned}  $$

In a general structured model of the form (1) with *n*_*a*_ distinct classes, $n_{a}^{2}$ transmission terms are required. However, one transmission term is available at most for each class. The typical way to address this lack of specificity is to constrain the structure of the transmission matrix and/or to use prior knowledge of social mixing behavior. For an age-structured model, we assume that the transmission rates are proportional to the rates of social contact, which can be estimated from contact patterns. A large multi-country population-based survey conducted in Europe as a part of the POLYMOD [9] enables us to implement this approach. The transmission is modeled as the product of the contact rate in the survey and an age-specific proportionality factor to account for characteristics related to susceptibility and infectiousness, which are not captured by contact rates. This leads to

$$ \beta_{{ij}}=\left\{\begin{array}{ll} q_{i} c_{{ij}} & \quad i=j \\ \sigma c_{{ij}} & \quad i\neq j \end{array}\right. $$

where *c*_*ij*_ is the contact rate and *q*_*i*_ and *σ* are proportionality factors.

Based on the age-structured SIR model (1), the reproduction number can be calculated by following Driessche and Watmough [10]. It is the spectral radius of the next generation matrix *M* where

$$M_{{ij}} =\frac{\beta_{{ij}} S_{i}(t)}{\gamma_{i}}. $$

The details are given in Appendix.

### Region-structured model

The second mechanism incorporates a heterogeneous population based on regions to account for the wave of the pH1N1 pandemic. We denote the number of susceptible and infected individuals within the *i*^*th*^ region by *S*_*i*_ and *I*_*i*_, respectively. Let *β*_*ij*_ refer to the transmission from the *j*^*th*^ subgroup to the *i*^*th*^ subgroup and *β* =[*β*_*ij*_] denote the transmission matrix. In the same manner as the age-structured model, we have the following system of differential equations.

2 $$  \begin{aligned} S_{i}^{\prime}(t) &= -\sum_{j=1}^{n_{r}} \beta_{{ij}} S_{i}(t)I_{j}(t) \\ I_{i}^{\prime}(t) &= \sum_{j=1}^{n_{r}} \beta_{{ij}} S_{i}(t)I_{j}(t) - \gamma_{i} I_{i}(t). \end{aligned}  $$

We assume that transmission rates between distinct regions in the region-structured model can be expressed as the frequency of transportations multiplied by a region-specific proportionality factor. The transportation information was extracted from the highway portal site and Kakao map for number of buses and highway traffic, respectively [11, 12]. Let the number of buses and highway traffic from region *j* to region *i* be denoted by *w*_*i,j*_ and *W*_*i,j*_, respectively. Note that *w* is symmetric because the bus route is circular, although *W* is not necessarily. The transmission rate can be written as

$$ \beta_{{ij}}=\left\{\begin{array}{ll} q_{i} & \quad i=j \\ q_{i} \sigma_{l} w_{{ij}} + q_{i} \sigma_{g} W_{{ij}} & \quad i\neq j \end{array}\right. $$

where *q*_*i*_ is the proportionality factor, and *σ*_*l*_ are *σ*_*g*_ can be chosen such that they balance the weight between different types of transportations.

The same argument as that presented in the age-structured model gives the expression of the effective reproduction number *R*_*t*_, which is the spectral radius of the following next generation matrix

$$\left[ \frac{\beta_{{ij}} S_{i}(t)}{\gamma_{i}}\right]. $$

### Study subjects and parameter estimation

Study subjects were patients who were prescribed antiviral agents from the national stockpile from August 21, 2009 to April 30, 2010. Because of mandatory antiviral agent management program during study period, all patients who were prescribed antiviral agent were included in this study. The data employed to estimate the parameters are the daily number of incident patients in Fig. 1. It was estimated based on the aggregation of prescribed antiviral agents from deidentified database. This study was approved by the Institutional Review Board (IRB) of Yonsei University Health System. Since this study used retrospective data and the study subjects were anonymized, the IRB waived the requirement for written consent from the patients.

Our goal is to estimate the optimal parameters that provide the states that are best fit to the given data. This section briefly reviews the parameter estimation technique of the least squares method. In general, parameter estimation is conducted by minimizing the cost function, which measures the difference between the model prediction and observation. The simplex algorithm proposed by John Nelder and Roger Mead is applied to solve the optimization problem [13]. Let ***θ*** be the parameter set and time points *t*_*j*_(*j*=1,...,*N*) are uniformly distributed with daily time step. The data vector ***y***_*j*_(*j*=1,...,*N*) denotes the number of cases at time *t*_*j*_. It is a scalar for basic SIR model, but it is a vector structured by age and region for age-stratified and region-stratified models, respectively. For example, the ***y***_*j*_ is a column vector of length 15 for the age-structured model. We recast the mathematical model as

$$ \boldsymbol{x}^{\prime}(t)= \boldsymbol{g}\left(t, \boldsymbol{x}(t), \boldsymbol{\theta}\right), $$

and assume a statistical model for measurement of the form

$$ \boldsymbol{y}_{j} = \boldsymbol{f}\left(t_{j};\boldsymbol{\theta}\right) + \varepsilon_{j}, \qquad j=1,\cdots,N $$

where ***f***(*t*_*j*_;***θ***) is the model prediction at time *t*_*j*_ with parameter ***θ*** and the measurement error $\varepsilon _{j} \sim \mathcal {N}\left (0, a^{2}\right)$. The least squares estimator can be obtained by minimizing the following cost function over the given parameter space *Ω*_*θ*_ [14]:

3 $$ \sum_{j=1}^{N} \left[\boldsymbol{y}_{j} - \boldsymbol{f}\left(t_{j};\boldsymbol{\theta}\right)\right]^{T}\left[\boldsymbol{y}_{j} - \boldsymbol{f}\left(t_{j};\boldsymbol{\theta}\right)\right]  $$

The parameter sets to be estimated for the basic SIR model, age-structured model and region-structured model, are

$$\begin{aligned} \boldsymbol{\theta}_{\text{basic}} &= \left\{\beta, \gamma\right\} \text{and initial values of {S} and {I},}\\ \boldsymbol{\theta}_{\text{age}} &= \left\{q_{i}, \sigma, \gamma_{i} \text{ for} i=1, 2,\cdots, n_{a}\right\} \text{and initial values of \(S_{i}\) and \(I_{i}\),} \\ \boldsymbol{\theta}_{\text{region}} &= \left\{q_{i}, \sigma_{l}, \sigma_{g}, \gamma_{i} \text{ for} i=1, 2,\cdots, n_{r} \right\} \text{and initial values of \(S_{i}\) and \(I_{i}\)}\\ \end{aligned} $$

where *n*_*a*_ and *n*_*r*_ denote the number of age groups and regions, respectively.

## Results

### Time-dependent reproduction number

**Fig. 3:**
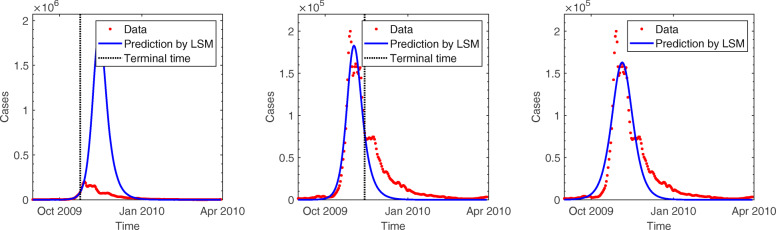
Comparison of pH1N1 incidence data with predictions of least squares method (LSM) using the basic SIR model (top and bottom left): The red dots show the number of new cases per day and the blue line presents the predicted number of cases. Terminal time of data used for estimation is displayed by a black dotted vertical bar. In each figure, the end of time period was set at October 24, 2009, November 14, 2009, and March 30, 2010

**Fig. 4:**
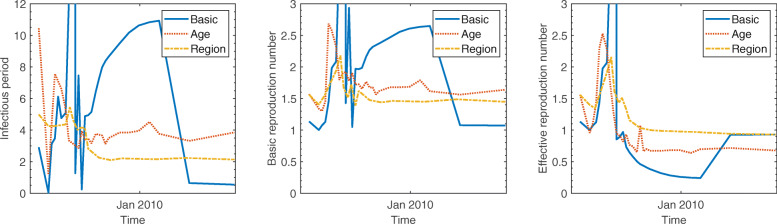
Estimated parameters and prediction using three different models: The x-axis denotes the terminal time *t* of data collection for which we have computed three different quantities as time passes. The duration of infectious period 1/*γ*(*t*) and transmission rate *β*(*t*) were estimated by fitting the model prediction to the number of cases as the data collection period increased. Then the basic reproduction number was obtained by computing *β*(*t*)*N*/*γ*(*t*) with the estimated parameters, where *N* represented the size of total population. And the effective reproduction number was calculated through *β*(*t*)*S*(*t*)/*γ*(*t*) using the model prediction *S*(*t*) with estimated parameters

We illustrate the proposed methodology and investigate its performance by applying it to 2009 incidence data of pH1N1 in South Korea. This section presents the results of the estimation obtained by applying the least squares method to basic SIR, age- and region-structured models. In each experiment, data with different time periods by varying the terminal time are tested to determine the earliest stage of the epidemic sufficient to provide a reasonable estimation. Figure 3 displays the predicted incidence based on the basic SIR model compared with the observed data. Predictions using data only during the initial growth phase cannot effectively exhibit the dynamics and substantially overestimate the spread of the infection. The results of simulation improved after the peak of the epidemic, and the wave is roughly generated at a later stage. However, the simple SIR model does not provide a reasonable estimation of parameters. The estimated values of *γ* and *R*_0_ demonstrate a large variation and remains outside of the feasible range for the influenza, regardless of the time period for data in Fig. 4. The plausible reason for this involves the model assumptions that are too simple to capture the underlying mechanisms.

**Fig. 5:**
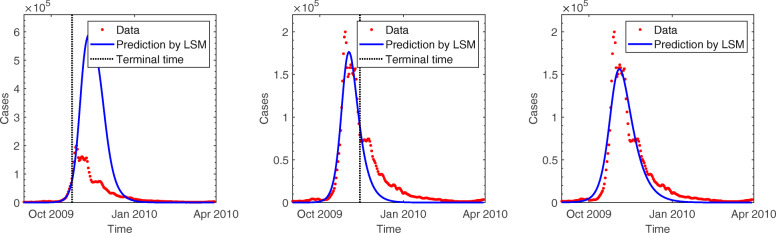
Comparison of pH1N1 incidence data with predictions of least squares method (LSM) using age-structured SIR model (top and bottom left): The red dots show the number of new cases per day, and the blue line presents the predicted number of cases. Terminal time of data used for estimation is displayed by a black dotted vertical bar. In each figure, the end of time period was set at October 24, 2009, November 14, 2009, and March 30, 2010

For the age-structured SIR model (1), the total population is split into 14 subgroups of 5-year age bands and one with 70 years and older (i.e., 0-4, 5-9,..., 65-69, 70 ^+^). This incorporates a heterogeneous population into the model in order to reflect different transmission rates in each age group. In Fig. 5, the outbreaks are simulated using data during various time periods in the same manner as mentioned above. Results of both the models show similar trends as long as the terminal time is earlier than mid-November when the gentle growth begins during the decline stage. Additionally, as the growth begins to decline, the age-structured SIR model fits the incidence data better than the basic SIR model. In Fig. 4, the reproduction number starts increasing in early October, peaks at 2.5 on October 17, and then decreases to unity at the end of October. Real-time estimation demonstrated that the effective reproduction number rose sharply during mid-October when the number of patients increased dramatically. The reproduction number fell below unity at the end of October and stayed lower than unity indicating that the epidemic starts decreasing, which is consistent with the incidence data. In the age-stratified model, heterogeneity was incorporated by WAIFW matrix where the transmission was assumed to be proportional to the contacts. The effective contacts were measured by POLYMOD contact survey, which showed a clear evidence for an age-dependency in contact patterns. Taking heterogeneous mixing into the model enabled better description of the dynamics, because the trend in behavior was consistent with the demographic characteristics of cases (as shown in Table 1). Estimated parameters are possible indicators to determine the feasibility of models. Incorporation of the age structure allows for robust estimation of parameters, while the basic SIR model provides estimated values beyond the reasonable range with severe fluctuation in Fig. 4. Table 2 summarizes the parameter estimates using three different models. The estimated duration of infectious period using age-structured model is around 3.8. The reproduction number was estimated to be 1.6 which is similar to those obtained in Mexico, the United States, New Zealand, Peru, and Chile [2, 15–18].

**Table 2:** Estimated parameters using three different models

	Basic SIR	Age-structured SIR	Region-structured SIR
*R*_0_	1.0722	1.6398	1.4487
Infectious period	0.5462	3.8539	2.1359

**Fig. 6:**
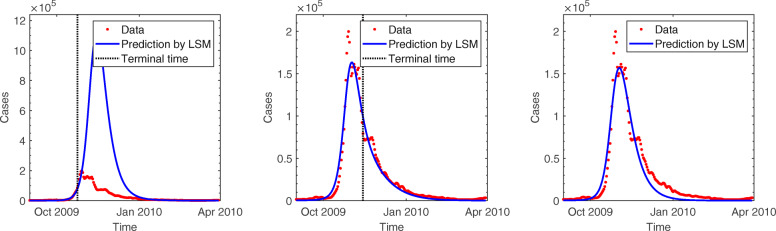
Comparison of pH1N1 incidence data with the predictions of least squares method (LSM) obtained using the region-structured SIR model (top and bottom left): The red dots show the number of new cases per day, and the blue line presents the predicted number of cases. The terminal time of data used for estimation is displayed by a black dotted vertical bar. In each figure, the end of time period was set at October 24, 2009, November 14, 2009, and March 30, 2010

The general characteristics of regional difference led us to consider a second type of heterogeneity in the model. The nation is split into 252 in the region-structured model (2), where the transmission rates are implemented based on transportation patterns. Figure 6 compares the predicted cases based on the region-structured SIR model with the observed data over the course of the epidemic. As it was discussed in the previous experiment, it is not earlier than the epidemic peak for estimation to start adjusting to outbreak data. Since this outbreak, the incidence data is well described in the form of the characteristic exponential rise, turnover, and decline pattern predicted by the process model. The estimated duration of infectious period using region-structured model is around 2.1 and the reproduction number was estimated to be 1.4 (Table 2). The time-dependent effective reproduction number is also illustrated in Fig. 4, which demonstrates a pattern similar to that obtained using the age-structured SIR model.

**Fig. 7:**
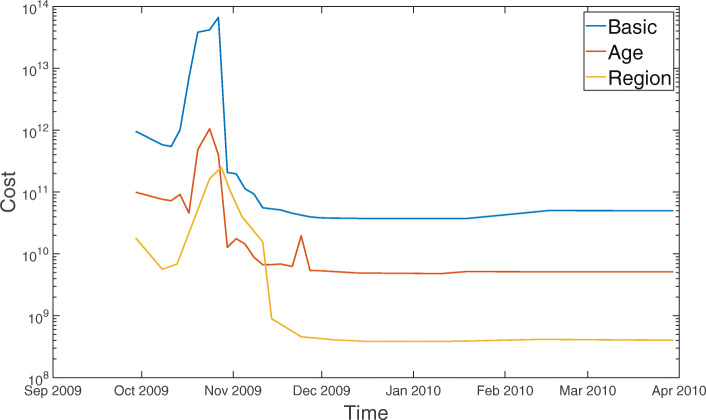
Residual sum of squares (RSS) which measures the goodness-of-fit in least squares estimation using three different models as time passes: The time average values of RSS for the basic, age-structured and region-structured models are 1.097×10^11^,1.787×10^9^, and 7.023×10^8^, respectively

Estimated duration of infectious period and reproduction numbers using three different models are compared in Fig. 4. Values of the cost function defined by (3) are also provided in Fig. 7, which shows the goodness-of-fit in the order of region-structured, age-structured and simple SIR model.

### Age-specific and region-specific reproduction numbers

**Fig. 8:**
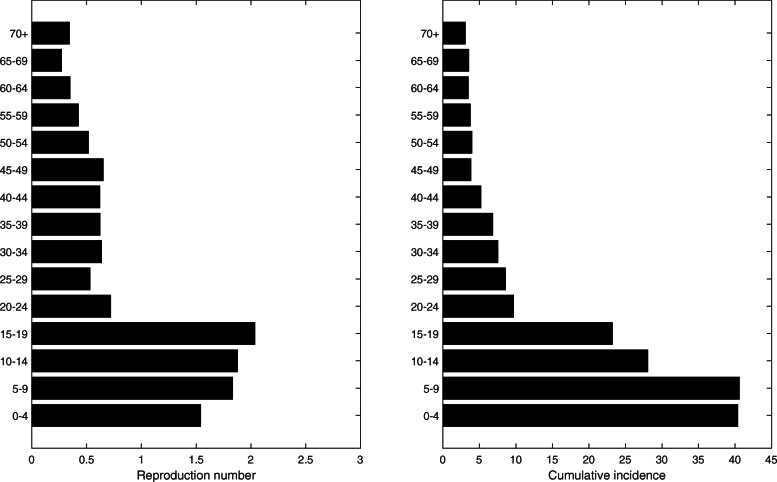
Age-specific reproduction number (left) and cumulative incidence for each age group (right)

It is widely known that the transmission is considerably different among various age groups. We also observe from the pH1N1 epidemic data that the incidence rate is higher in children and students than in other age groups (Table 1). Estimates of the age-specific reproduction number help in clarifying the role of each age group in the transmission dynamics and in suggesting guidelines for effective targeting intervention strategies. The estimated age-specific reproduction numbers are displayed in Fig. 8. The result is closely related to the cumulative incidence for each age group because it is often the contact rate within the same age group is higher than with other groups.

**Fig. 9:**
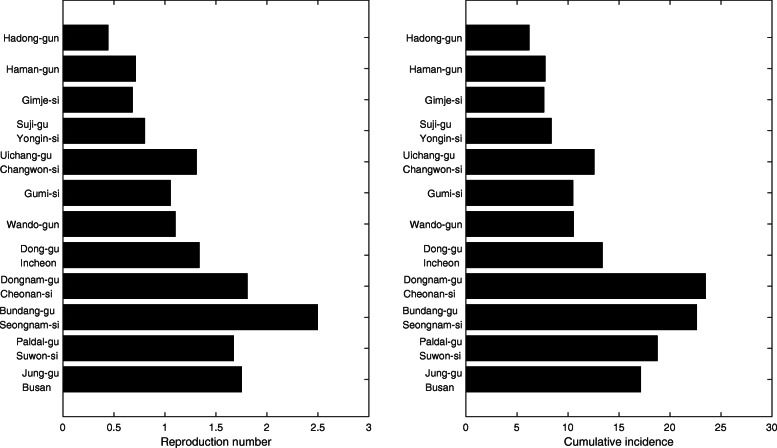
Region-specific reproduction number (left) and cumulative incidence for each region (right)

The incidence rate is higher in urban areas than in rural areas, and the highest in the national capital and the south-eastern region, as shown in Fig. 2. We estimated the region-specific reproduction number and observed that it is more than two in some areas and less than one in the others (Fig. 9). This is consistent with regions having larger cumulative incidence with a similar argument regarding contact patterns to age-specific cases.

## Discussion

An estimation of reproduction numbers is crucial because it provides a measure of the transmission potential when an infection is spreading throughout a population. The reproductive numbers in the early phase of Influenza A(H1N1)pdm09 have been estimated in several countries with different settings, yielding median 1.46 and range 1.0–3.6 [19]. Many of these studies focused on cases confirmed in the early stage of the pandemic. Because laboratory tests focused on severe cases and there are possible changes in laboratory testing and notification rates, the number of confirmed cases does not necessarily represent the underlying epidemic. It also does not reflect the dynamics during the period of the highest level of activity, which is the winter in temperate climates. Some studies used the number of cases from sentinel surveillance that is much less than the actual number of influenza patients. It is necessary to estimate the reproductive number using the number of all the patients throughout a pandemic, including the period with the highest level of activity. In this study, the reproductive number was estimated based on the national data of incidence deduced from antiviral agent prescription in South Korea during the pandemic.

We discussed parameter estimation methodologies based on deterministic SIR models that included age or spatial structures with the main aim being to estimate the effective reproduction numbers, *R*_0_ and *R*_*t*_. There could be many modelling choices to compute these important epidemiological parameters, including simple SIR model with time varying parameters [20, 21] or renewal equations [22, 23]. We proposed one possible approach to introduce population heterogeneity since we observed demographic and regional characteristics of incidence data. Age-structured and region-structured models were employed to describe the underlying epidemic process, in particular. To avoid exacerbating non-identifiability problem by increasing the complexity of a model, age- and region-specific data were used to estimate parameters for age- and region-structured model, respectively. And contact measurements of POLYMOD contact matrix (for age-structured model) and transportation information (for region-structured model) have been incorporated to compensate the discrepancy in the increased dimension of transmission parameters and the data. Nevertheless, we are aware that the complexity of this type of model can leave some problems of non-identifiability.

The proposed mechanisms were applied to influenza A(H1N1)pdm09 in South Korea to compute the time-dependent effective reproduction numbers. Real-time estimation showed that the reproduction number started increasing in early October, peaked at 2.5 on October 17, and then decreased to unity at the end of October. The effective number rose sharply during the mid-October when the number of patients increased dramatically. The reproduction number fell below unity at the end of October and remained lower that unity, indicating that the epidemic starts decreasing, which is consistent with the incidence data.

Subsequently, age-specific and region-specific basic reproduction numbers were estimated to account for the differences of incidence. We observe from the pH1N1 epidemic data that the incidence rate is higher in children and students than in other age groups. The estimated age-specific reproduction numbers agree with the cumulative incidence for each age group because the mixing is assortative. The incidence rate is higher in urban areas than in rural areas, highest in the national capital and in the south-eastern region. We estimated the region-specific reproduction number whose trend is similar to the number of cases in each region. Estimates of the age-specific and region-specific reproduction number help to predict the transmission dynamics, and to suggest guidelines for effective targeting intervention strategies.

This study has both limitations and strengths. First, the number of cases is estimated from the amount of prescribed antiviral agents assuming the time lag between symptom onset and antiviral agent prescription, the proportion of prescription and the proportion of pH1N1 confirmation among AFRI patients. Also, vaccination is not considered in the model. However, the effect of vaccination on the transmission of pH1N1 may have been insignificant because the vaccination for general group was initiated in January 2010. The effective contacts were employed from POLYMOD contact survey, which possibly yields discrepancy in mixing pattern of Korea [24]. The use of POLYMOD as well as the potential non-identifiability of complex models are limitations of this study. We will be able to improve the outcome as we gather more information, because additional knowledge is required to achieve a better result.

On the contrary, the present research has its strengths compared to previous studies. The reproduction number was estimated based on the national level antiviral agent prescription data in South Korea throughout the pandemic including the period of the highest level of activity. The real-time estimation incorporating population structures can be used to predict the disease dynamics, thereby providing guidelines for the optimal implementation of preventive measures, such as school closing and distribution of antiviral agents.

## Conclusions

Numerical results reveal that the introduction of heterogeneity into the population and sufficient data to represent general characteristics of dynamics are essential to the robust estimation of parameters. Real-time estimation showed that the reproduction number started increasing in early October, peaked on October 17, and then decreased to fell below unity at the end of October, which is consistent with the incidence data. The estimated age- and region-specific reproduction numbers are also consistent with cumulative incidence for corresponding groups.

## Appendix

The reproduction number for the age-structured SIR model (1), can be calculated following the approach of Driessche and Watmough [10]. Let $\mathcal {F}_{i}$ be the new infections and $\mathcal {V}_{i}$ be the transitions of *i*^*th*^ compartment, then

4 $$ \mathcal{F}_{i} = S_{i}\left(q_{i} c_{{ii}}I_{i} + \sum_{j\neq i} \sigma c_{{ij}}I_{j}\right)  $$

and

5 $$ \mathcal{V}_{i} = \gamma I_{i}.  $$

for *i*=1,⋯,*n*_*a*_.

Subsequently, the derivatives of $\mathcal {F}=[\mathcal {F}_{i}]$ and $\mathcal {V}=[\mathcal {V}_{i}]$ are

$$\begin{aligned} F &= \left[\begin{array}{cccc} S_{1} q_{1} c_{11} & S_{1} \sigma c_{12} & \cdots & S_{1} \sigma c_{1n_{a}} \\ S_{2} \sigma c_{21} & S_{1} q_{2} c_{22} & \cdots & S_{2} \sigma c_{2n_{a}} \\ \vdots & \vdots & \ddots & \vdots \\ S_{n_{a}} \sigma c_{n_{a}1} & S_{n_{a}} \sigma c_{n_{a}2} & \cdots & S_{n_{a}} q_{n_{a}} c_{n_{a}n_{a}} \end{array}\right],\\ \quad V &= \left[\begin{array}{llll} \gamma & 0 & \cdots & 0 \\ 0 & \gamma & \cdots & 0 \\ \vdots & \vdots & \ddots & \vdots \\ 0 & 0 & \cdots &\gamma \end{array}\right], \end{aligned} $$

respectively, and the next generation matrix is

6 $$ FV^{-1} = \frac{1}{\gamma} \left[\begin{array}{cccc} S_{1} q_{1} c_{11} & S_{1} \sigma c_{12} & \cdots & S_{1} \sigma c_{1n_{a}} \\ S_{2} \sigma c_{21} & S_{1} q_{2} c_{22} & \cdots & S_{2} \sigma c_{2n_{a}} \\ \vdots & \vdots & \ddots & \vdots \\ S_{n_{a}} \sigma c_{n_{a}1} & S_{n_{a}} \sigma c_{n_{a}2} & \cdots & S_{n_{a}} q_{n_{a}} c_{n_{a}n_{a}} \end{array}\right].  $$

Thus, the reproduction number is the spectral radius of *FV*^−1^ and the age-specific reproduction number is the column sum of *FV*^−1^ corresponding to the age of interest.

## Data Availability

All data generated or analysed during this study are included in the published article [8]. The datasets used and/or analysed during the current study are available from the corresponding author on reasonable request.

## Abbreviations

AFRI: Acute febrile respiratory illness
IRB: Institutional review board
WAIFW: Who acquires infection from whom
LSM: Least squares method
RSS: Residual sum of squares
